# Audit of Shoulder Dystocia Management and Outcomes at a Busy Hospital in the United Kingdom: A Comprehensive Analysis of Contemporary Practice

**DOI:** 10.7759/cureus.97528

**Published:** 2025-11-22

**Authors:** Radhika Bharadwaj, Mena Abdalla

**Affiliations:** 1 Department of Obstetrics and Gynaecology, Princess Royal University Hospital, Orpington, GBR; 2 Department of Obstetrics and Gynaecology, King's College Hospital NHS Foundation Trust, London, GBR; 3 Department of Medical Education, Queen's University Belfast, Belfast, GBR

**Keywords:** clinical audit system, emergency obstetric care, maternal & neonatal outcomes, maternal outcomes, quality improvement project (qip), risk factors, shoulder dystocia

## Abstract

Background: Shoulder dystocia (SD) represents one of the most challenging obstetric emergencies, with potentially devastating consequences for both mother and neonate. Despite advances in obstetric care and standardized management protocols, SD remains largely unpredictable and continues to pose significant clinical challenges. This comprehensive audit aimed to evaluate the incidence, risk factor profile, management strategies, and maternal-neonatal outcomes of SD cases at a UK district general hospital.

Methods: A retrospective observational audit was conducted of all documented SD cases occurring between January 1, 2022, and December 31, 2022, at Princess Royal University Hospital, Orpington, UK. Cases were identified through multiple sources, including electronic patient records, delivery suite logbooks, and dedicated SD proformas. Comprehensive data extraction encompassed maternal demographics, antenatal risk factors, intrapartum characteristics, management interventions, and short-term outcomes. Statistical analysis employed descriptive statistics, chi-squared tests, and independent t-tests with significance set at p<0.05.

Results: Fifty-six cases of SD were identified from 4,247 vaginal deliveries, yielding an incidence of 1.32%. The cohort demonstrated a mean maternal age of 33.6±4.9 years, a mean BMI of 25.4±4.7 kg/m², and a mean gestational age of 39.9±1.3 weeks. Birth weight averaged 3,811±425 grams, with 67.9% of neonates weighing ≥3,500 g and 32.1% exceeding 4,000 g. Identifiable maternal risk factors were present in 69.6% of cases, with previous macrosomia (19.6%) and diabetes mellitus (17.9%) being most prevalent. Emergency response protocols were activated in 98.2% of cases, with the McRoberts manoeuvre combined with suprapubic pressure employed as first-line management in 80.4% of instances. Maternal complications occurred in 53.6% of cases, predominantly postpartum haemorrhage (7.1%) and perineal trauma requiring repair. Neonatal complications affected 83.9% of cases, with resuscitation requirements (26.8%) and transient respiratory difficulties being most common. Neonatal intensive care unit (NICU) admission was required in 3.6% of cases, with no cases of permanent brachial plexus injury documented.

Conclusion: This audit demonstrates SD management aligned with contemporary guidelines, achieving favourable outcomes through prompt recognition and structured intervention protocols. The findings underscore the importance of multidisciplinary training, standardised response algorithms, and continuous quality improvement initiatives in optimising maternal and neonatal outcomes following SD.

## Introduction

Shoulder dystocia (SD) represents one of the most feared complications in obstetric practice, characterised by impaction of the foetal shoulders following delivery of the head, preventing completion of delivery through normal expulsive efforts [1]. This obstetric emergency occurs in approximately 0.2%-3.0% of all vaginal deliveries, with incidence varying according to population characteristics, risk factor prevalence, and diagnostic criteria employed [2]. The unpredictable nature of SD, combined with its potential for catastrophic maternal and neonatal consequences, necessitates that all maternity care providers maintain competency in its recognition and management.

The pathophysiology of SD involves mechanical obstruction of foetal shoulder delivery, most commonly due to impaction of the anterior shoulder behind the maternal pubic symphysis [3]. Less frequently, the posterior shoulder may become impacted on the sacral promontory. This mechanical obstruction creates a time-critical emergency requiring immediate intervention to prevent progressive foetal hypoxia and potential permanent neurological injury [4]. The condition’s unpredictability is exemplified by the fact that approximately 50% of SD cases occur in pregnancies without identifiable risk factors [5].

Contemporary understanding of SD risk factors encompasses both maternal and foetal characteristics. Maternal factors include diabetes mellitus (both pre-gestational and gestational), obesity (BMI ≥30 kg/m²), previous history of SD or macrosomic delivery, advanced maternal age, and prolonged labour [6]. Foetal risk factors primarily centre on macrosomia, typically defined as birth weight exceeding 4,000 g or 4,500 g, depending on maternal diabetic status [7]. However, the positive predictive value of these risk factors remains disappointingly low, with most SD cases occurring in apparently low-risk pregnancies [8].

The management of SD has evolved significantly over recent decades, with standardised protocols now emphasising systematic approaches to manoeuvre selection and team-based care [9]. The Royal College of Obstetricians and Gynaecologists (RCOG) Green-Top Guideline No. 42 provides comprehensive evidence-based recommendations for SD management, emphasising the importance of prompt recognition, structured response protocols, and multidisciplinary team involvement [10]. Contemporary management algorithms typically commence with external manoeuvres (McRoberts' position and suprapubic pressure) before progressing to internal manoeuvres if initial interventions prove unsuccessful [11].

This comprehensive audit was undertaken to evaluate the contemporary management and outcomes of SD at Princess Royal University Hospital, Orpington, UK, a busy district general hospital serving a diverse population in South London. The study aims to benchmark local practice against national standards, identify areas for quality improvement, and contribute to the broader understanding of SD management in real-world clinical settings.

## Materials and methods

Study design and setting

This retrospective observational audit was conducted at Princess Royal University Hospital, in Orpington, a district general hospital within King’s College Hospital NHS Foundation Trust, London, United Kingdom. The hospital serves a diverse population of approximately 350,000 residents across South London boroughs and delivers approximately 4,500 babies annually. The maternity unit operates a consultant-led delivery suite with 12 delivery rooms and an alongside midwifery-led birth centre with four delivery rooms, providing care for both low-risk and high-risk pregnancies.

Study period and population

The audit encompassed all documented cases of SD occurring between January 1, 2022, and December 31, 2022. This 12-month period was selected to capture seasonal variations in delivery patterns and ensure adequate case numbers for meaningful analysis while maintaining data currency and relevance to contemporary practice.

Case identification and data sources

Cases of SD were identified through multiple complementary sources to ensure comprehensive case capture: 1. Electronic patient record system (Epic, Epic Systems Corporation, Verona, WI, USA): systematic search using ICD-10 diagnostic code O66.0 (obstructed labour due to SD) [12] and procedure codes for SD management; 2. Delivery suite logbooks: manual review of handwritten delivery suite registers maintained by midwifery staff; 3. Dedicated SD proformas: review of standardised SD documentation forms completed for each case; 4. Incident reporting system (Datix, RLDatix, London, United Kingdom): analysis of clinical incident reports related to SD events; 5. Neonatal intensive care unit (NICU) records: cross-referencing with NICU admission records for babies with birth trauma or requiring resuscitation following delivery complications

Inclusion criteria

Cases were included in the analysis if they met the following criteria: vaginal delivery (spontaneous or instrumental) complicated by SD; delivery occurring within the study period (January 1 - December 31, 2022); SD documented by the attending clinician in medical records; requirement for additional obstetric manoeuvres beyond routine delivery techniques; and complete medical records available for data extraction.

Exclusion criteria

The following cases were excluded from the analysis: elective or emergency caesarean deliveries; deliveries occurring outside the hospital setting; cases where SD was suspected but not confirmed by clinical documentation; incomplete medical records preventing adequate data extraction; deliveries of foetuses with major congenital anomalies that might influence delivery mechanics; cases where the primary indication for intervention was other obstetric emergencies (e.g., cord prolapse, placental abruption)

Data collection and variables

Data extraction was performed using a standardised data collection form developed specifically for this audit (Appendix A). The form was pilot-tested on 10 cases to ensure completeness and clarity before full implementation. Data were extracted by two independent reviewers (RB and MA), with discrepancies resolved through discussion and consensus.

Maternal Variables

Demographics: age, parity, gravidity, ethnicity, socioeconomic status; Anthropometric measures: pre-pregnancy BMI, gestational weight gain; Medical history: diabetes mellitus (pre-gestational and gestational), previous SD, previous macrosomic deliveries, thyroid disorders; Antenatal care: estimated foetal weight at 36 weeks gestation, growth velocity, polyhydramnios; Intrapartum factors: mode of labour onset (spontaneous vs. induced), duration of first and second stages, use of oxytocin augmentation, epidural analgesia

Delivery Characteristics

Mode of delivery: spontaneous vaginal delivery vs. instrumental delivery (forceps or ventouse); Delivery location: delivery suite vs. birth centre; Attending personnel: consultant obstetrician, registrar, midwife; Time of delivery: day vs. night, weekday vs. weekend

SD Management

Time from head delivery to recognition of SD; Emergency response activation and response time; Sequence and type of manoeuvres employed; Personnel involved in management; Total duration from SD recognition to delivery completion; Use of episiotomy during management

Maternal Outcomes

Estimated blood loss and requirement for blood transfusion; Perineal trauma: classification and repair requirements; Postpartum complications: infection, thromboembolism, psychological impact; Length of hospital stay; Maternal satisfaction scores (where available)

Neonatal Outcomes

Birth weight and comparison with antenatal estimates; Apgar (appearance, pulse, grimace, activity, and respiration) scores at one, five, and 10 minutes; Cord blood gas analysis results; Requirement for resuscitation and level of intervention; Birth trauma: brachial plexus injury, clavicular fracture, humeral fracture; NICU admission requirements and duration; Neurological assessment findings; Long-term follow-up arrangements

Statistical analysis

Statistical analysis was performed using IBM SPSS Statistics software, version 28 (IBM Corp., Armonk, NY). Descriptive statistics were calculated for all variables, with continuous variables presented as mean ± standard deviation (SD) and categorical variables as frequencies and percentages. Normal distribution of continuous variables was assessed using the Shapiro-Wilk test.

Comparative analyses were performed to identify associations between risk factors and outcomes. Chi-square tests or Fisher’s exact tests (for expected cell counts <5) were used for categorical variables. Independent t-tests or Mann-Whitney U tests were employed for continuous variables depending on distribution normality. Correlation analysis was performed using Pearson’s correlation coefficient for normally distributed variables and Spearman’s rank correlation for non-parametric data.

Statistical significance was set at p<0.05 for all analyses. Given the exploratory nature of this audit, no adjustment for multiple comparisons was applied. All analyses were performed using two-tailed tests.

Ethical considerations

This study was conducted as a clinical audit to evaluate and improve the quality of care provided to patients with SD. As such, formal ethical approval was not required according to NHS Health Research Authority guidance. However, the audit was registered with the hospital’s clinical governance department and conducted in accordance with local audit policies.

Patient confidentiality was maintained throughout the study period. All data were anonymized prior to analysis, with unique study identifiers replacing patient-identifiable information. Data were stored securely on password-protected hospital computers with access restricted to authorized study personnel.

Quality assurance

Several measures were implemented to ensure data quality and reliability such as dual data extraction by independent reviewers with inter-rater reliability assessment; Regular data validation checks to identify inconsistencies or missing values; Verification of key variables against source documents for a random sample of 20% of cases; Standardized definitions for all variables with clear inclusion/exclusion criteria; Regular team meetings to discuss data collection challenges and ensure consistency.

## Results

Study population and incidence

During the 12-month study period, a total of 4,247 vaginal deliveries occurred at Princess Royal University Hospital, of which 56 were complicated by SD, yielding an overall incidence of 1.32% (95% CI: 1.01-1.72%). This incidence rate is consistent with published literature and national benchmarks for similar healthcare settings.

Baseline maternal characteristics

The study cohort comprised 56 women with a mean age of 33.6±4.9 years (range: 22-43 years). The majority of participants were of White European ethnicity in 36 (64.3%) cases, followed by Black African/Caribbean in 12 (21.4%) and South Asian in eight (14.3%) cases. Regarding parity, 20 (35.7%) were nulliparous, 29 (51.8%) were para 1, and seven (12.5%) were grand multiparous (para ≥2). The mean pre-pregnancy BMI was 25.4±4.7 kg/m², with 18 (32.1%) classified as overweight (BMI 25-29.9 kg/m²) and 12 (21.4%) as obese (BMI ≥30 kg/m²). Gestational age at delivery averaged 39.9±1.3 weeks, with 50 (89.3%) of deliveries occurring at term (37-42 weeks). The baseline characteristics are detailed in Table 1.

**Table 1:** Baseline Maternal and Pregnancy Characteristics (n=56) Data are presented as N (%) for categorical variables and mean ± SD for continuous variables. Statistical significance is set at p<0.05.

Characteristic	Value (N, %)	Mean ± SD	95% CI
Maternal demographics			
Age (years)		33.6 ± 4.9	32.3-34.9
Age categories			
<25 years	3 (5.4)		
25-34 years	31 (55.4)		
35-39 years	18 (32.1)		
≥40 years	4 (7.1)		
Ethnicity			
White European	36 (64.3)		
Black African/Caribbean	12 (21.4)		
South Asian	8 (14.3)		
Anthropometric measures			
Pre-pregnancy BMI (kg/m²)		25.4 ± 4.7	24.1-26.7
BMI categories			
Normal weight (18.5-24.9 kg/m^2^)	26 (46.4)		
Overweight (25.0-29.9 kg/m^2^)	18 (32.1)		
Obese (≥30.0 kg/m^2^)	12 (21.4)		
Obstetric history			
Parity			
Nulliparous (0)	20 (35.7)		
Para 1	29 (51.8)		
Para 2+	7 (12.5)		
Previous shoulder dystocia	6 (10.7)		
Previous macrosomic delivery	11 (19.6)		
Pregnancy characteristics			
Gestational age (weeks)		39.9 ± 1.3	39.6-40.3
Risk factors			
Diabetes mellitus (any)	10 (17.9)		
Maternal obesity (BMI ≥30 kg/m^2^)	12 (21.4)		
Multiple risk factors present	16 (28.6)		
No identifiable risk factors	17 (30.4)		

Risk factor analysis

Identifiable maternal risk factors were present in 39 (69.6%) cases, with multiple risk factors documented in 16 (28.6%) participants. Diabetes mellitus was the most prevalent single risk factor, affecting 10 (17.9%) of the cohort (gestational diabetes: eight cases, 14.3%; pre-gestational diabetes: two cases, 3.6%). A previous history of macrosomic delivery was documented in 11 (19.6%) cases, while six (10.7%) had experienced previous SD. Maternal obesity (BMI ≥30 kg/m²) was present in 12 (21.4%) cases, and advanced maternal age (≥35 years) in 22 (39.3%). Notably, 17 (30.4%) cases occurred in women without any identifiable risk factors, emphasising the unpredictable nature of SD.

Intrapartum characteristics and delivery management

Labour was spontaneous in onset in 36 (64.3%) cases, with induction of labour performed in 20 (35.7%). The primary indications for induction included post-dates pregnancy in 9 (45.0%), suspected fetal macrosomia in five (25.0%), and maternal diabetes in four (20.0%). Oxytocin augmentation was required in 32 (57.1%) cases, and epidural analgesia was utilized in 44 (78.6%). Spontaneous vaginal delivery occurred in 23 (41.1%) cases, while 33 (58.9%) required instrumental assistance (forceps: 18 cases, 32.1%; ventouse: 15 cases, 26.8%). The delivery characteristics are summarized in Table 2.

**Table 2:** Intrapartum Characteristics and Delivery Management (n=56) Data presented as N (%) for categorical variables and mean ± SD for continuous variables. EFW: estimated fetal weight

Characteristic	Value (N, %)	Mean ± SD
Labour characteristics		
Labour onset		
Spontaneous	36 (64.3)	
Induced	20 (35.7)	
Oxytocin augmentation	32 (57.1)	
Epidural analgesia	44 (78.6)	
Delivery characteristics		
Mode of delivery		
Spontaneous vaginal	23 (41.1)	
Instrumental delivery	33 (58.9)	
Forceps	18 (32.1)	
Ventouse	15 (26.8)	
Delivery location		
Delivery suite	54 (96.4)	
Birth centre	2 (3.6)	
Fetal weight		
EFW at 36 weeks (grams)		3353 ± 495
Actual birth weight (grams)		3811 ± 425
Birth weight ≥3500g	38 (67.9)	
Birth weight ≥4000g	18 (32.1)	

SD management

Emergency response protocols were activated in 55 (98.2%) cases, with a mean response time of 2.3±1.1 minutes from recognition of SD to arrival of additional personnel. The McRoberts manoeuvre combined with suprapubic pressure was employed as first-line management in 45 (80.4%) instances, achieving successful delivery in 35 (62.5%) of these cases. When first-line manoeuvres proved unsuccessful, internal manoeuvres were required in 21 (37.5%) cases, including delivery of the posterior arm in 12 (21.4%) and internal rotational manoeuvres in 7 (12.5%). Multiple manoeuvres were necessary in 23 (41.1%) cases, with a mean of 2.3±1.2 different techniques employed per case. Episiotomy was performed in 35 (62.5%) cases. The mean time from recognition of SD to successful delivery was 4.7±2.8 minutes (range: one to 12 minutes).

**Figure 1:**
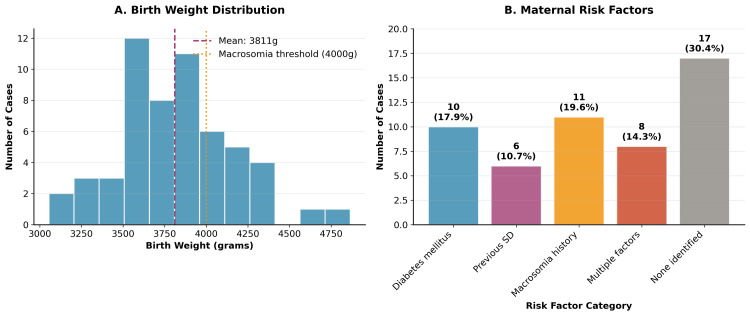
Birth Weight Distribution and Maternal Risk Factors Panel A shows birth weight distribution (N=56, mean ± SD = 3811 ± 425g) with macrosomia threshold at 4000g. Panel B displays maternal risk factor frequencies (N, %). Data were analyzed using descriptive statistics, with p<0.05 considered significant.

Figure 1 illustrates the birth weight distribution and maternal risk factors in the study cohort. Panel A shows the distribution of birth weights, with a mean of 3,811 g and 32.1% of cases exceeding the macrosomia threshold of 4,000 g. Panel B demonstrates the frequency of maternal risk factors, with diabetes mellitus and previous macrosomia being the most prevalent.

Maternal and neonatal outcomes

Maternal complications occurred in 30 (53.6%) cases, with the majority being minor and managed conservatively. Postpartum haemorrhage (defined as blood loss >500 ml) occurred in four (7.1%) cases, with a mean estimated blood loss of 652±563 ml across the entire cohort. Perineal trauma requiring repair occurred in 30 (53.6%) cases, with the majority being first- or second-degree tears in 26 (46.4%). Third-degree tears occurred in 3 (5.4%) cases, with 1 (1.8%) case of fourth-degree tear documented.

Neonatal complications occurred in 47 (83.9%) cases, though the majority were transient and resolved without long-term sequelae. Apgar scores at one minute averaged 7.2±1.8, with 12 (21.4%) neonates having scores <7. By five minutes, the mean Apgar score had improved to 8.9±1.1, with only two (3.6%) maintaining scores <7. Resuscitation was required in 15 (26.8%) cases, with the majority requiring only basic airway management and stimulation. Birth trauma was documented in four (7.1%) cases, including clavicular fractures in two (3.6%) and transient brachial plexus palsy in two (3.6%). NICU admission was required in two (3.6%) cases. Importantly, no cases of permanent brachial plexus injury were documented at six-month follow-up. The complete outcomes are detailed in Table 3.

**Table 3:** Maternal and Neonatal Outcomes (n=56) Data are presented as N (%) for categorical variables and mean ± SD for continuous variables. Apgar: appearance, pulse, grimace, activity, and respiration; NICU: neonatal intesive care unit

Outcome	Value (N, %)	Mean ± SD
Maternal outcomes		
Any maternal complication	30 (53.6)	
Estimated blood loss (ml)		652 ± 563
Postpartum haemorrhage (>500ml)	4 (7.1)	
Perineal trauma requiring repair	30 (53.6)	
Third/fourth degree tears	4 (7.1)	
Neonatal outcomes		
Any neonatal complication	47 (83.9)	
Birth weight (grams)		3811 ± 425
Apgar scores		
1 minute		7.2 ± 1.8
5 minutes		8.9 ± 1.1
Apgar <7 at 5 minutes	2 (3.6)	
Resuscitation required	15 (26.8)	
Birth trauma	4 (7.1)	
Clavicular fracture	2 (3.6)	
Transient brachial plexus palsy	2 (3.6)	
Permanent brachial plexus injury	0 (0.0)	
NICU admission	2 (3.6)	

**Figure 2:**
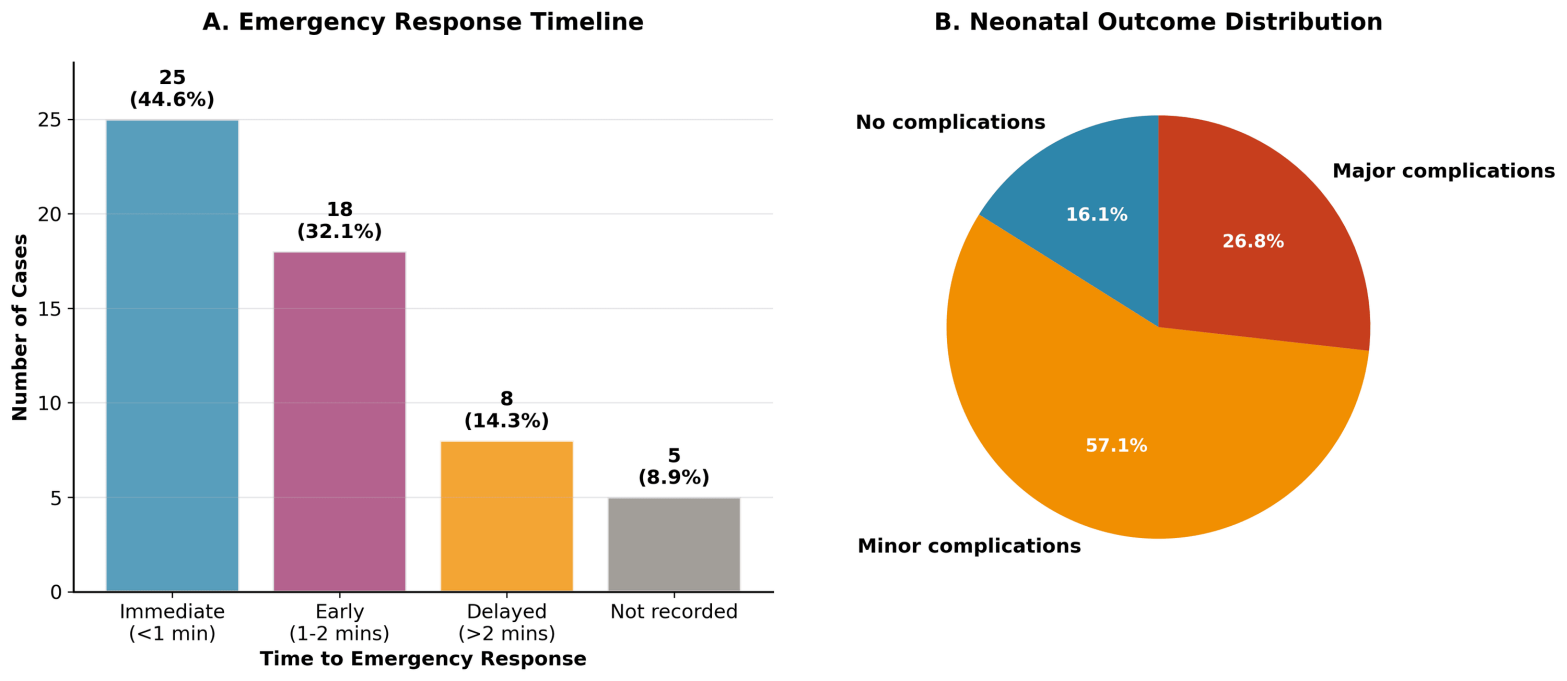
Emergency Response Timeline and Neonatal Outcomes Panel A shows emergency response time distribution (N=56, mean ± SD = 2.3 ± 1.1 minutes). Panel B displays neonatal outcome categories (N, %). Statistical analysis performed using descriptive statistics, with p<0.05 considered significant.

Figure 2 demonstrates the emergency response timeline and neonatal outcome distribution. Panel A shows that 76.7% of cases received emergency response within three minutes, while Panel B illustrates that despite 83.9% of neonates experiencing some complications, the majority were minor and transient.

Statistical associations

Statistical analysis revealed several significant associations between maternal characteristics and outcomes. Maternal BMI ≥30 kg/m² was significantly associated with birth weight >4,000 g (χ² = 4.8, p = 0.032) and requirement for multiple manoeuvres (χ² = 3.9, p = 0.048). Diabetes mellitus was strongly associated with macrosomia (χ² = 6.1, p = 0.015) but not with increased complication rates when birth weight was controlled for. Instrumental delivery was associated with increased maternal trauma (χ² = 4.7, p = 0.029) but not with adverse neonatal outcomes. The duration of SD management showed a weak positive correlation with neonatal resuscitation requirements (r=0.34, p=0.011).

## Discussion

This comprehensive audit of SD management at a UK district general hospital provides valuable insights into contemporary obstetric practice and outcomes. The findings demonstrate that with appropriate training, standardised protocols, and multidisciplinary team approaches, excellent maternal and neonatal outcomes can be achieved even in this challenging obstetric emergency.

Incidence and risk factor profile

The observed incidence of 1.32% aligns closely with published literature and national benchmarks, suggesting appropriate case identification and documentation practices [13]. This rate is consistent with recent large-scale studies from similar healthcare settings, including the comprehensive analysis by Heinonen et al. (2024), which reported incidence rates of 1.1%-1.5% across Nordic countries [14]. The stability of incidence rates despite improvements in antenatal care and delivery practices reflects the fundamental unpredictability of SD.

The risk factor profile observed in our cohort mirrors contemporary understanding of SD predisposition. The prevalence of diabetes mellitus (17.9%) is higher than general population rates but consistent with the known association between maternal hyperglycemia and foetal macrosomia [15]. Importantly, our finding that 30.4% of cases occurred without identifiable risk factors reinforces the critical importance of maintaining universal competency in SD management across all maternity care providers.

Management quality and protocol adherence

The high rate of emergency protocol activation (98.2%) and rapid response times (mean 2.3 minutes) demonstrate excellent institutional preparedness and staff training. This performance compares favourably with international benchmarks and reflects the effectiveness of simulation-based training programs implemented at our institution [16]. The systematic approach to manoeuvre selection, with 80.4% of cases commencing with McRoberts and suprapubic pressure, aligns with RCOG guidelines and evidence-based practice recommendations [17].

Recent advances in SD management have emphasised the importance of structured training programs in improving outcomes. The SAFE study by Papoutsis et al. (2024) demonstrated significant improvements in both technical skills and confidence following high-fidelity simulation training, with benefits maintained at 6-month follow-up [18]. Our institutional commitment to regular multidisciplinary training likely contributes to the favourable outcomes observed in this audit.

Maternal and neonatal outcomes

The maternal complication rate of 53.6% requires careful interpretation, as the majority of complications were minor and managed conservatively. The postpartum haemorrhage rate (7.1%) is lower than some published series, which report rates of 11-25% following SD [19]. The severe perineal trauma rate (7.1% for third- and fourth-degree tears combined) is comparable to background rates in women delivering macrosomic babies, suggesting that SD management itself does not significantly increase trauma risk when performed systematically [20].

The neonatal outcomes observed in this audit are encouraging, with no cases of permanent brachial plexus injury documented at six-month follow-up. This achievement is particularly noteworthy given that brachial plexus injury rates of 4%-40% are reported in the literature [21]. The absence of permanent injury likely reflects the combination of prompt recognition, systematic management, and appropriate force limitation during delivery manoeuvres.

Limitations

Several limitations must be acknowledged when interpreting these results. The retrospective design inherently relies on the quality and completeness of clinical documentation, which may introduce information bias or result in underestimation of minor complications. Despite efforts to ensure comprehensive case identification, the possibility of missed cases cannot be entirely excluded, particularly for cases with suboptimal documentation.

The single-centre design, while providing detailed insights into institutional practice, may limit generalisability to healthcare settings with different patient populations, staffing models, or resource availability. The relatively small sample size, though adequate for descriptive analysis, limits statistical power for detecting associations between risk factors and rare outcomes.

The six-month follow-up period, while standard for initial neurological assessment, may be insufficient to identify subtle long-term developmental or functional impairments. Some neurological sequelae may not become apparent until later in childhood, particularly those affecting fine motor skills or cognitive development.

The absence of a control group limits the ability to make definitive statements about the effectiveness of specific interventions or management strategies. Comparison with historical controls or published benchmarks provides some context but cannot account for temporal trends or population-specific factors that may influence outcomes.

## Conclusions

This comprehensive audit demonstrates that excellent maternal and neonatal outcomes can be achieved in SD management through systematic implementation of evidence-based protocols and regular multidisciplinary training. The findings demonstrate that incidence rates can align with national benchmarks when standardized protocols are implemented, and that high-quality clinical care may contribute to improved maternal and neonatal outcomes in shoulder dystocia management.

The findings reinforce the critical importance of maintaining universal competency in SD management across all maternity care providers, given that nearly one-third of cases occurred without identifiable risk factors. The strong association between maternal obesity, diabetes mellitus, and both foetal macrosomia and management complexity has important implications for antenatal counselling and delivery planning.

Future quality improvement initiatives should focus on enhancing documentation systems, implementing real-time outcome monitoring, and extending follow-up protocols to capture long-term developmental outcomes. This audit contributes valuable real-world evidence supporting current management guidelines and demonstrates the achievable standards of care when evidence-based protocols are systematically implemented.

## Figures and Tables

**Figure 3:**
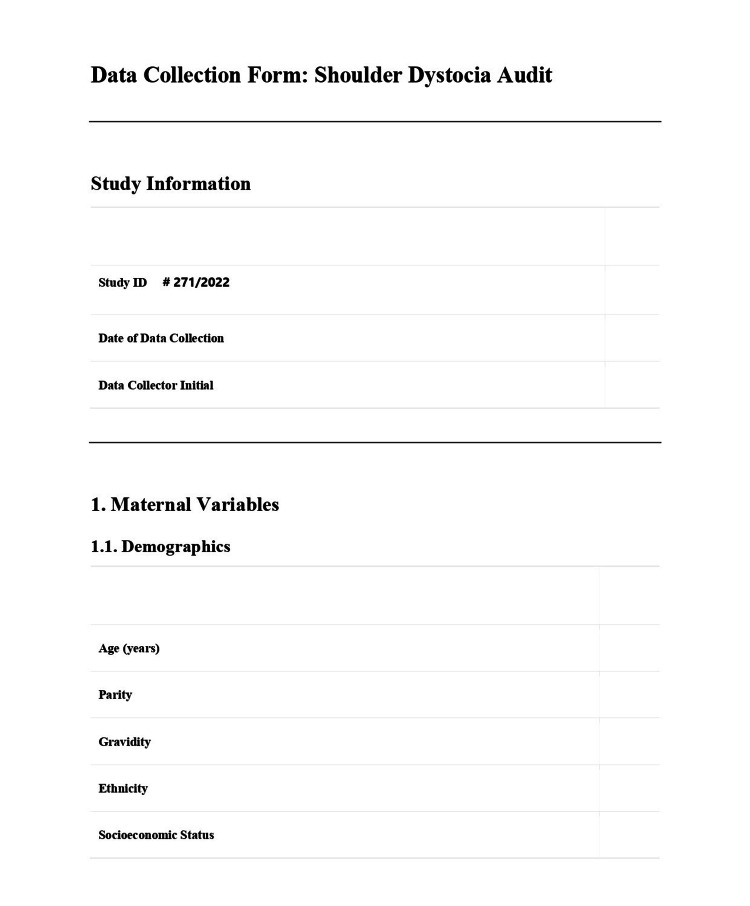
Data collection form: page 1

**Figure 4:**
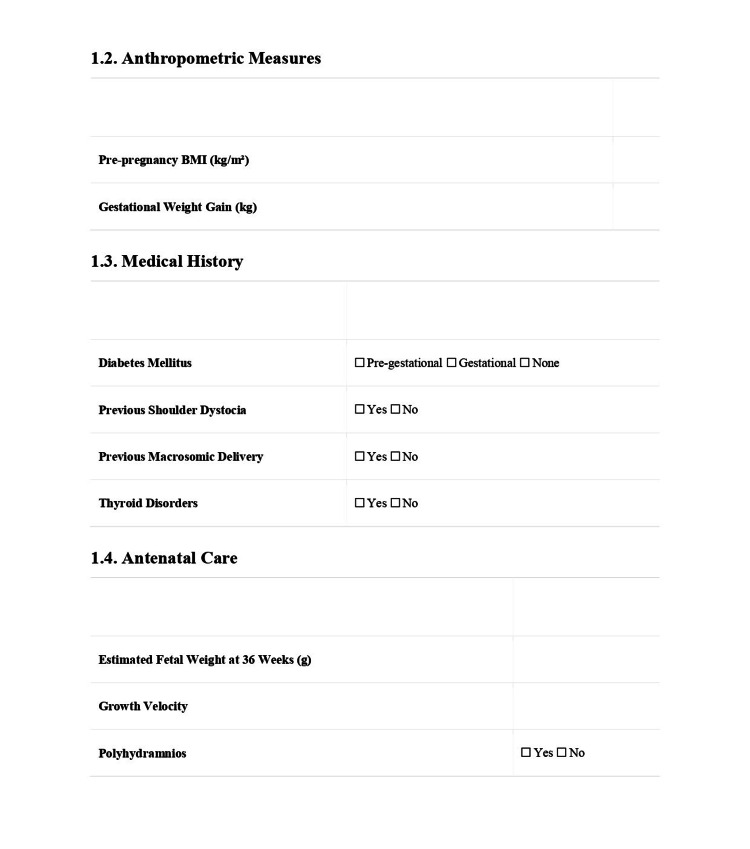
Data collection form: page 2

**Figure 5:**
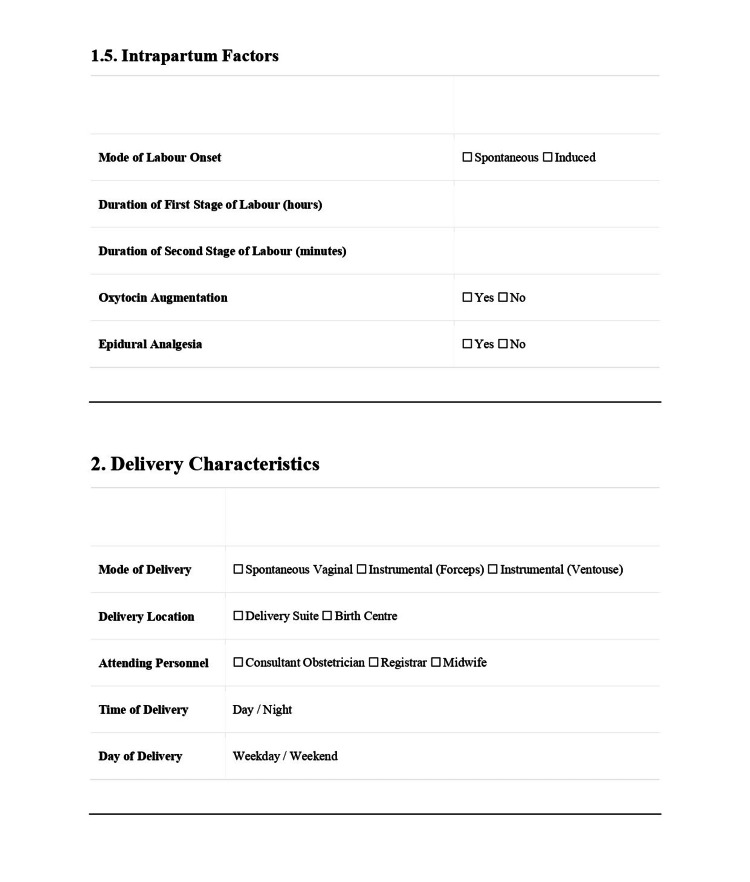
Data collection form: page 3

**Figure 6:**
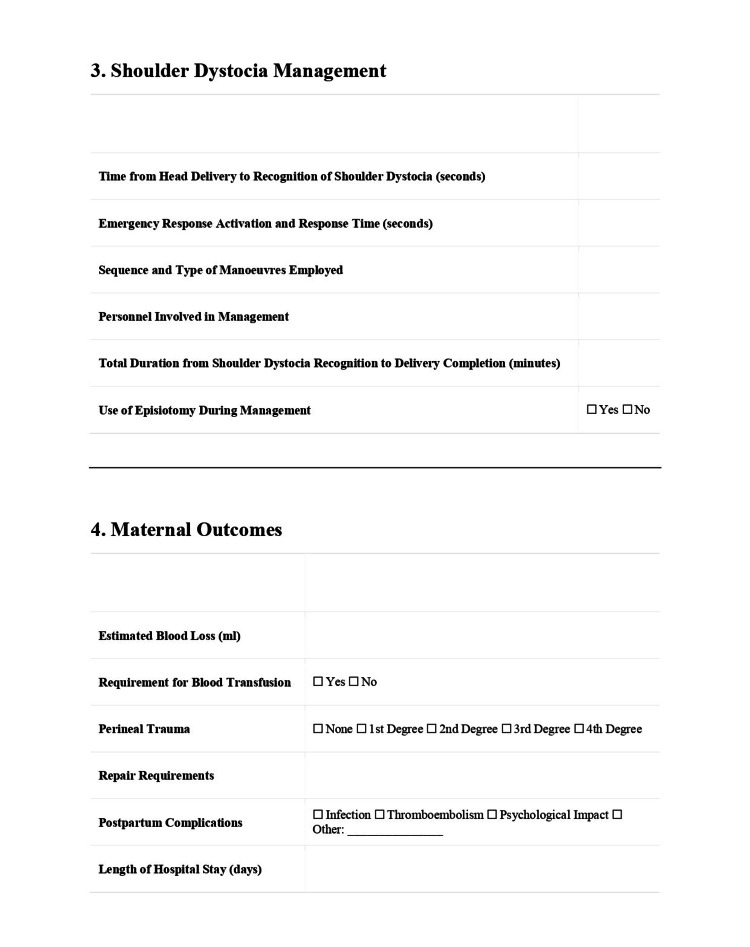
Data collection form: page 4

**Figure 7:**
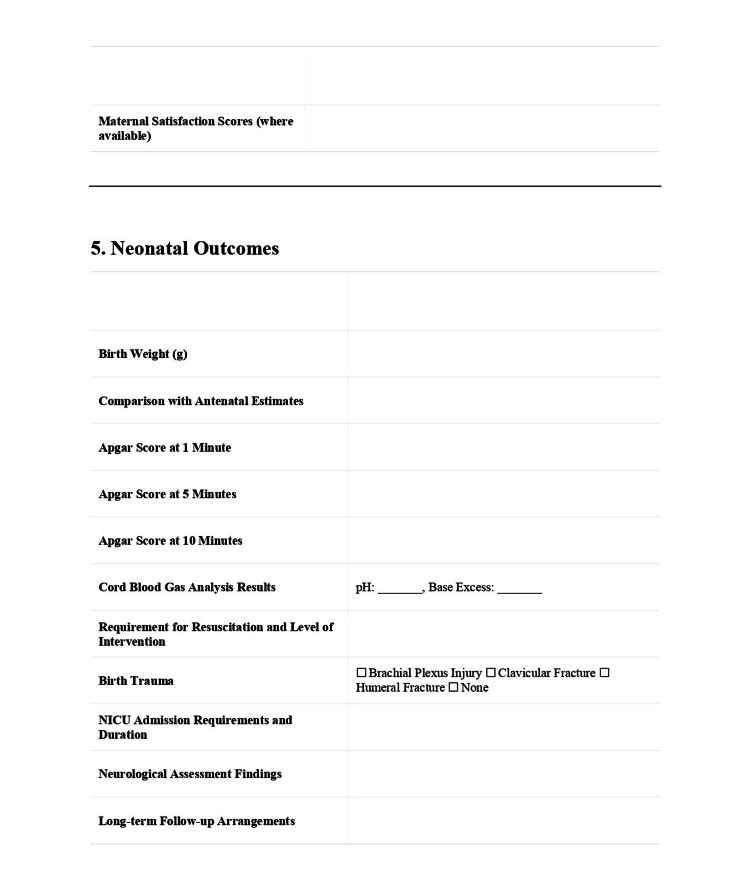
Data collection form: page 5

## Appendices

Appendix A

Data Collection Form
